# Applications of Novel Microscale and Nanoscale Materials for Theranostics: From Design to Clinical Translation

**DOI:** 10.3390/pharmaceutics16101339

**Published:** 2024-10-18

**Authors:** Mengxiang Tian, Bingzhi Dong, Weiqi Li, Liying Wang, Hong Yu

**Affiliations:** 1 Department of General Surgery, Sir Run Run Shaw Hospital, School of Medicine, Zhejiang University, Hangzhou 310058, China; 12218560@zju.edu.cn (M.T.); 22218120@zju.edu.cn (B.D.); 3170104490@zju.edu.cn (W.L.); 2 Provincial Key Laboratory of Precise Diagnosis and Treatment of Abdominal Infection, Sir Run Run Shaw Hospital, School of Medicine, Zhejiang University, Hangzhou 310016, China

**Keywords:** micro-nanomaterial drug delivery systems, design principles of micro-nanomaterial, therapeutic and diagnostic potential, clinical translation

## Abstract

The growing global prevalence of chronic diseases has highlighted the limitations of conventional drug delivery methods, which often suffer from non-specific distribution, systemic toxicity, and poor bioavailability. Microscale and nanoscale materials have emerged as innovative solutions, offering enhanced targeting, controlled release, and the convergence of therapeutic and diagnostic functions, referred to as theranostics. This review explores the design principles, mechanisms of action, and clinical applications of various novel micro- and nanomaterials in diseases such as cancer, cardiovascular disorders, and infectious diseases. These materials enable real-time monitoring of therapeutic responses and facilitate precision medicine approaches. Additionally, this paper addresses the significant challenges hindering clinical translation, including biocompatibility, potential toxicity, and regulatory issues. Ongoing clinical trials demonstrate the potential of nanomaterials in theranostic applications, but further research is needed to overcome the barriers to widespread clinical adoption. This work aims to contribute to the acceleration of integrating nanomedicine into clinical practice, ultimately enhancing the efficacy and safety of therapeutic interventions.

## 1. Introduction

As the global population continues to age and the incidence of chronic diseases rises significantly, improving therapeutic outcomes and minimizing drug-related side effects have become pivotal challenges in modern medical research [1]. Conventional drug delivery methods frequently encounter substantial limitations, including non-specific drug distribution, heightened systemic toxicity, and suboptimal bioavailability [2]. The non-targeted dispersion of drugs often leads to their unintended accumulation in healthy tissues, resulting in significant adverse effects and constraining the therapeutic dosages that can be safely administered. Furthermore, the rapid metabolism of many drugs within the body prevents them from maintaining sufficient therapeutic concentrations, thereby diminishing their overall efficacy [3]. In response to these challenges, researchers are increasingly focused on developing advanced drug delivery systems that offer enhanced precision and efficiency, with the aim of improving drug targeting, enabling controlled release, and consequently achieving superior therapeutic outcomes while reducing systemic toxicity.

Micro-nanomaterials have emerged as highly promising candidates for next-generation drug delivery systems due to their distinctive physicochemical properties, such as nanoscale dimensions, surface modifications, and customizable functional characteristics. These properties enable these materials to overcome biological barriers, achieve prolonged systemic circulation, and deliver drugs with high specificity to targeted sites, thereby markedly increasing drug concentrations at disease sites [4,5]. Additionally, the emergence of micro-nanomaterials for theranostic applications introduces an innovative paradigm, wherein therapeutic and diagnostic functions are integrated within a single platform. The physicochemical properties of these materials not only enhance drug delivery but also enable simultaneous imaging and monitoring of disease progression, providing real-time feedback for personalized treatment strategies. These materials, designed for multifunctional roles, allow for precise targeting while offering diagnostic data through techniques like MRI, CT, or fluorescence imaging. This dual capability is crucial for precision medicine, particularly in oncology, where early detection and targeted treatment are paramount [6]. However, despite significant advances in preclinical research, the clinical translation of these micro-nanomaterial-based drug delivery systems remains fraught with challenges, including concerns regarding biocompatibility, potential toxicity, and complex in vivo pharmacokinetics [7,8].

This review aims to provide a comprehensive analysis of the applications of novel micro-nanomaterials in terms of their therapeutic and diagnostic potential. We also critically assess the key obstacles to their clinical translation and propose potential strategies to address these challenges, with the ultimate goal of advancing the practical implementation of micro-nanomaterials in medicine and accelerating their integration into clinical practice.

## 2. Design of Micro-Nanomaterial for Theranostics

Theranostics, which combines therapeutic and diagnostic functions in a single platform, represents a significant leap forward in personalized medicine. In the realm of micro-nanomaterials, the design of drug delivery systems now extends beyond traditional drug carriers [9]. These materials are engineered to facilitate both the precise delivery of therapeutic agents and the simultaneous real-time monitoring of treatment efficacy through diagnostic imaging modalities. This dual capability is vital in improving patient outcomes, particularly in fields like oncology, cardiovascular disease, and infectious diseases [10]. The following sections discuss how micro-nanomaterials are being specifically tailored to meet the demands of theranostic systems.

### 2.1. Classification and Characteristics of Micro-Nanomaterials for Theranostic Applications

Micro-nanomaterials for theranostics are categorized based on their composition and functionality into organic, inorganic, and composite materials. Each class provides unique advantages for integrating therapeutic and diagnostic capabilities (Table 1).

Organic materials encompass polymer nanoparticles, liposomes, dendrimers, and peptide-based nanomaterials. Polymeric nanoparticles such as poly(lactic-co-glycolic acid) (PLGA) have shown great potential in drug delivery; in theranostics, PLGA nanoparticles can be co-loaded with therapeutic agents and imaging probes, such as fluorescent markers or MRI contrast agents, allowing for simultaneous drug delivery and diagnostic imaging [11]. Liposomes are spherical vesicles with a phospholipid bilayer that mimics cell membranes, making them highly biocompatible. Liposomes can encapsulate therapeutic drugs as well as contrast agents, such as gadolinium for MRI, enabling real-time tracking of drug distribution. In cancer theranostics, liposomes are functionalized with targeting ligands that bind to tumor-specific markers, improving drug delivery precision while enhancing the imaging of tumor sites [12]. Dendrimers are hyper-branched macromolecules with a well-defined architecture, providing multiple sites for drug and imaging agent attachment. Their high loading capacity makes them ideal for theranostics. For instance, dendrimers conjugated with chemotherapy drugs and fluorescent dyes can target cancer cells while enabling real-time monitoring of therapeutic efficacy [13].

Inorganic materials include gold nanoparticles, superparamagnetic iron oxide nanoparticles (SPIONs), and mesoporous silica nanoparticles (MSNs). Gold nanoparticles are widely used in theranostics due to their unique optical properties. In photothermal therapy (PTT), AuNPs absorb near-infrared light, generating heat to ablate tumors, while their optical properties allow for enhanced imaging contrast. AuNPs can be conjugated with drugs or imaging agents, providing both treatment and diagnostic functions [14]. SPIONs are employed in magnetic resonance imaging (MRI) due to their magnetic properties. In theranostics, SPIONs are functionalized with therapeutic agents for drug delivery, and their magnetic properties allow for precise tracking through MRI. SPION-based theranostic systems are particularly effective in detecting and treating metastatic cancer [15]. MSNs offer high surface area and tunable pore sizes, allowing for the simultaneous loading of drugs and imaging agents. In theranostics, MSNs can deliver chemotherapeutic agents while also enhancing fluorescence imaging or MRI, providing dual functionality for cancer treatment [16].

Composite materials combine the advantages of organic and inorganic materials, offering enhanced drug delivery, imaging capabilities, and multifunctionality. For instance, silica-coated gold nanoparticles integrate the photothermal properties of gold with the high loading capacity of silica, enabling combined photothermal therapy and imaging. These hybrid materials are especially promising in theranostics as they facilitate both the therapeutic and diagnostic capabilities within a single platform [17,18].

### 2.2. Functionalization of Materials and Optimization of Theranostic Systems

Functionalization is critical in theranostics to ensure targeted delivery, controlled drug release, and enhanced imaging capabilities. Modifying the surface of micro-nanomaterials with targeting ligands, such as antibodies or peptides, enhances their ability to home in on specific disease sites, while the inclusion of imaging probes allows for real-time monitoring (Table 2).

#### 2.2.1. Targeting and Imaging

The integration of targeting and imaging technologies is a crucial aspect in optimizing theranostic systems. Through the synergistic action of targeting ligands and imaging probes, nanomaterials can not only precisely localize to pathological sites but also enable real-time imaging for monitoring, providing strong support for personalized therapy. The surface of nanomaterials can be functionalized with ligands that target specific receptors overexpressed on diseased cells, such as the folate receptor in cancer cells. In theranostic systems, this ensures precise drug delivery to the site of interest while also enhancing imaging contrast. For example, liposomes functionalized with folate and loaded with doxorubicin and a fluorescent dye allow for targeted cancer therapy and real-time fluorescence imaging [19,20]. Theranostic nanoparticles are functionalized with diagnostic probes like quantum dots or fluorophores for fluorescence imaging, or MRI contrast agents such as gadolinium. These materials provide visual feedback on drug distribution and efficacy during therapy, allowing for real-time adjustments in treatment [21].

#### 2.2.2. Stimuli-Responsive Systems

Stimuli-responsive micro-nanomaterials release their therapeutic payload in response to specific environmental triggers, such as pH, temperature, or enzymes, making them highly effective in theranostics. Tumor microenvironments often exhibit lower pH levels than healthy tissues. pH-sensitive nanoparticles can be designed to release drugs specifically in acidic environments, enhancing targeted drug delivery while minimizing systemic toxicity. These nanoparticles can also be loaded with imaging agents, providing feedback on drug release locations [22]. Enzyme-responsive materials are particularly useful in theranostics for diseases like cancer, where certain enzymes are overexpressed. These materials degrade in response to the target enzyme, releasing their therapeutic cargo. For example, matrix metalloproteinase (MMP)-responsive nanoparticles release drugs in the presence of MMPs, which are elevated in tumor environments [23].

#### 2.2.3. Smart Responsive Systems

Smart responsive micro-nanomaterials represent the forefront of theranostics, integrating external stimuli (e.g., light or magnetic fields) to control drug release and enhance imaging capabilities. Light-responsive nanoparticles, particularly those used in photothermal or photodynamic therapy, allow for precise control over drug release using external light sources. Gold nanoparticles are commonly employed in these systems due to their strong absorption in the near-infrared region. Upon light irradiation, these nanoparticles generate localized heat, disrupting cancer cells and enabling concurrent real-time imaging using optical methods [24]. Temperature-responsive materials undergo phase changes at specific temperatures, which can be exploited in hyperthermia treatments. These materials are often used in combination with MRI or ultrasound imaging to guide the treatment process, providing real-time feedback and enhancing therapeutic outcomes. This dual action is particularly useful in inflammation-related diseases [25].

### 2.3. Issues of Micro-Nanomaterials for Theranostic Applications

The combination of diagnostic and therapeutic capabilities within a single nanoparticle system—often referred to as a theranostic system—presents numerous technical and biological challenges. One of the fundamental limitations lies in the complexity of engineering nanoparticles that can fulfill both roles effectively without compromising either function. Nanoparticles designed for imaging purposes often require different materials and structures than those designed for therapeutic delivery, resulting in conflicting requirements [26]. Another critical issue concerns the pharmacokinetics and biodistribution of theranostic nanoparticles. Achieving a balance between diagnostic and therapeutic functions can lead to unpredictable biodistribution, where nanoparticles might accumulate more in certain tissues due to their diagnostic imaging properties rather than their therapeutic efficacy. This misbalance can limit the clinical applicability of the nanoparticles, as the required dose for therapeutic intervention may be lower or higher than that for imaging [27]. The last issue is the potential for toxicity, particularly with inorganic materials used in diagnostic applications (such as gold or iron nanoparticles) combined with organic drug delivery systems. The potential long-term accumulation of these inorganic materials can result in organ toxicity, as they may not be easily cleared from the body. Moreover, the need to optimize the size, surface charge, and hydrophilicity/hydrophobicity balance of nanoparticles to enhance both diagnostic and therapeutic efficacy complicates their design, as changes that favor one function can impair the other [28].

## 3. Applications of Micro-Nanomaterials in Therapeutics

Micro-nanomaterials, owing to their unique physicochemical properties, have demonstrated substantial potential in contemporary medicine, particularly in the treatment and diagnosis of various diseases. Their size effects, surface characteristics, and tunability enable them to overcome the limitations of traditional therapeutic methods, facilitating more precise and effective treatment strategies.

### 3.1. Applications in Cancer Therapy

**Figure 1:**
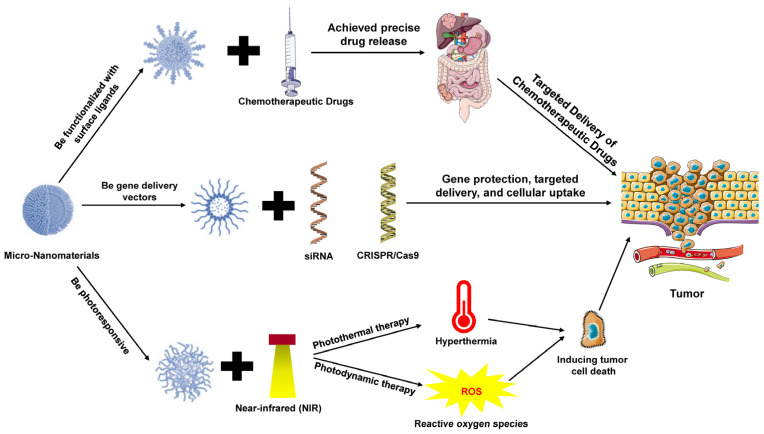
Applications of micro-nanomaterials in cancer therapy. Micro-nanomaterials improve cancer treatment by enabling targeted delivery of chemotherapeutic drugs, increasing drug concentration at tumor sites while minimizing damage to healthy tissues. These nanocarriers also enhance gene therapy by delivering siRNA or CRISPR/Cas9 for precise gene regulation. Additionally, micro-nanomaterials are integral to photothermal and photodynamic therapies, using light to induce localized tumor cell death, with high precision and minimal harm to surrounding tissues. These therapies can be combined with other treatments for improved outcomes.

Cancer treatment remains a significant challenge in the medical field. While traditional therapies such as surgery, chemotherapy, and radiotherapy have achieved varying degrees of success, their lack of specificity and associated high toxicity often lead to severe side effects [29]. The advent of micro-nanomaterials has introduced new avenues for cancer therapy, particularly in targeted drug delivery, gene therapy, and photothermal/photodynamic therapy, showing remarkable potential [30] (Figure 1).

#### 3.1.1. Targeted Delivery of Chemotherapeutic Drugs

Chemotherapy is a cornerstone of cancer treatment, yet traditional chemotherapeutic agents suffer from non-specific distribution within the body, often causing substantial damage to healthy tissues and resulting in severe side effects [31]. Micro-nanomaterials address this issue through targeted delivery strategies. Nanocarriers, such as liposomes, polymeric nanoparticles, and metallic nanoparticles, can be functionalized with surface ligands to facilitate efficient drug accumulation at tumor sites. By enhancing the enhanced permeability and retention (EPR) effect, these nanocarriers can localize at tumor sites for extended durations, significantly increasing the drug concentration in the diseased tissue while minimizing harm to healthy tissues [32,33]. Additionally, smart responsive nanomaterials can undergo structural changes within the tumor microenvironment to achieve precise drug release, further improving therapeutic efficacy.

#### 3.1.2. Gene Therapy

Gene therapy represents a novel approach to cancer treatment, particularly for cancer types that are challenging to treat with conventional methods. Micro-nanomaterials serve as effective gene delivery vectors, addressing critical challenges in gene therapy, such as gene protection, targeted delivery, and cellular uptake [34]. Common nanocarriers include polymeric nanoparticles, liposomes, and inorganic nanoparticles, which can deliver small interfering RNA (siRNA) or CRISPR/Cas9 gene-editing tools to achieve precise regulation of cancer-related genes [35]. For example, the delivery of siRNA can effectively silence oncogenes, thereby inhibiting tumor growth and metastasis [36,37].

#### 3.1.3. Photothermal Therapy and Photodynamic Therapy

Photothermal therapy (PTT) and photodynamic therapy (PDT) are innovative cancer treatment modalities based on micro- and nanomaterials. PTT employs photoresponsive nanomaterials to generate localized hyperthermia upon near-infrared light irradiation, directly inducing tumor cell death [38,39]. PDT, in contrast, involves the production of reactive oxygen species (such as singlet oxygen) by photosensitizers under specific wavelengths of light, leading to apoptosis in tumor cells [40,41]. The use of micro-nanomaterials in PTT and PDT offers high spatiotemporal selectivity, enabling precise tumor targeting through external light control, thereby significantly reducing damage to surrounding healthy tissues. Furthermore, PTT and PDT can be integrated with chemotherapy, immunotherapy, and other treatments to form multimodal therapeutic strategies, enhancing therapeutic outcomes and minimizing side effects [42,43].

### 3.2. Applications in Infectious Diseases

**Figure 2:**
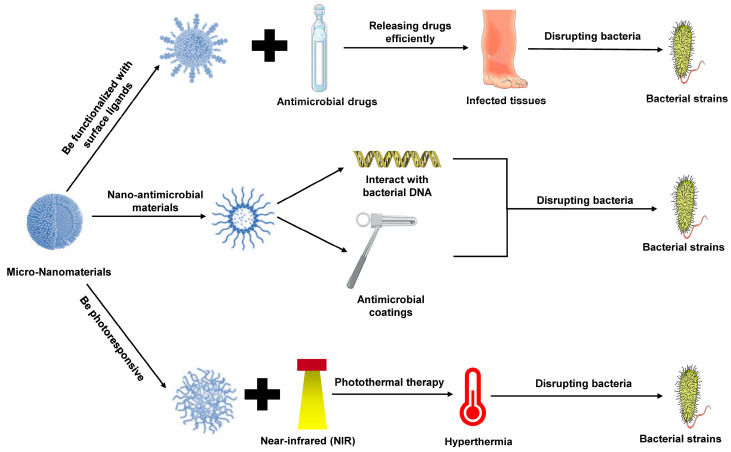
Applications of micro-nanomaterials in infectious diseases. Micro-nanomaterials enhance antimicrobial therapy by enabling targeted drug delivery, improving local drug concentration at infection sites while reducing systemic toxicity. Liposomes and functionalized nanocarriers efficiently release antibiotics at pathogen locations. Photothermal nanomaterials provide an alternative to combat antibiotic resistance by generating localized heat to kill resistant bacteria. Additionally, nanomaterials like silver nanoparticles and carbon nanotubes exhibit intrinsic antimicrobial properties and can enhance the efficacy of traditional antibiotics, offering novel strategies against multidrug-resistant strains and hospital-acquired infections.

Infectious diseases, particularly those caused by drug-resistant pathogens, present a significant threat to global public health. Traditional antibiotic treatments face unprecedented challenges due to the emergence of drug resistance [44]. Micro-nanomaterials offer new solutions for the treatment of infectious diseases, particularly through the targeted delivery of antimicrobial agents, the mitigation of drug resistance, and the development of novel antimicrobial therapies, demonstrating considerable application potential (Figure 2).

#### 3.2.1. Targeted Delivery of Antimicrobial Drugs

Micro-nanomaterials can significantly enhance the local concentration of antimicrobial drugs at infection sites, improving therapeutic outcomes through targeted delivery. Nanocarriers can be functionalized with specific ligands on their surface to recognize and target pathogens or infected tissues, releasing drugs efficiently at these locations. For instance, liposomes can encapsulate antibiotics and deliver them directly to the bacterial membrane at the infection site, thereby reducing non-specific distribution and the systemic toxicity of the drugs [45,46].

#### 3.2.2. Photothermal Therapy

Antibiotic resistance is a pressing issue in modern medicine. Micro-nanomaterials with photothermal properties provide a novel approach to overcoming this challenge. Photothermal materials can generate localized hyperthermia under near-infrared light irradiation, disrupting bacterial cell membranes and directly eliminating resistant bacterial strains [47,48,49]. These materials are effective against strains that traditional antibiotics cannot control, offering a promising alternative with reduced damage to healthy tissues.

#### 3.2.3. Nanomaterials as Antimicrobial Agents and Novel Antimicrobial Therapies

Beyond their role as delivery vehicles, certain nanomaterials exhibit intrinsic antimicrobial activity. For example, silver nanoparticles possess unique chemical properties that allow them to interact with bacterial proteins, DNA, and cell membranes, thereby inhibiting bacterial growth or inducing cell death [50,51]. Additionally, multifunctional nanomaterials, such as carbon nanotubes, have demonstrated potent antimicrobial effects [52]. When combined with traditional antibiotics, these nanomaterials can enhance antimicrobial efficacy and effectively combat multidrug-resistant strains. Moreover, nanomaterials can be employed as antimicrobial coatings on medical devices to prevent hospital-acquired infections. These novel antimicrobial therapies offer new possibilities for treating infectious diseases, especially in the context of increasing antimicrobial resistance [53,54].

### 3.3. Applications in Cardiovascular Diseases

**Figure 3:**
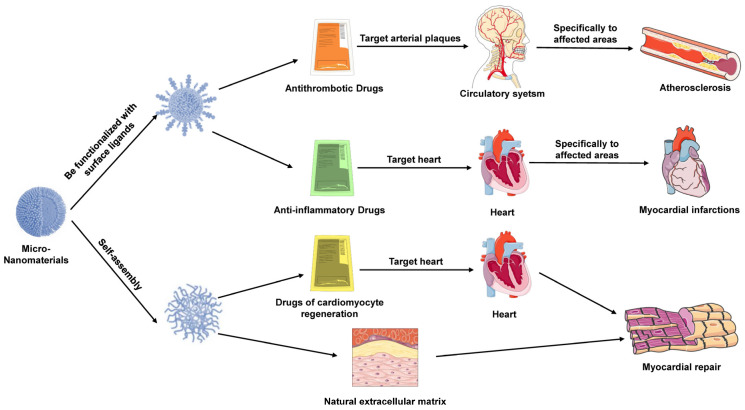
Applications of micro-nanomaterials in cardiovascular diseases. Micro-nanomaterials enhance cardiovascular therapies by enabling sustained, controlled drug release and targeted delivery. Systems like PLGA nanoparticles allow for continuous drug release, improving patient compliance, while magnetic nanoparticles offer controlled release via external fields. Targeted delivery of antithrombotic and anti-inflammatory drugs minimizes side effects and enhances efficacy. In atherosclerosis, functionalized nanomaterials target plaques, improving treatment precision. For myocardial repair, micro-nanomaterials deliver drugs and genes and form scaffolds that mimic the extracellular matrix, promoting cardiomyocyte regeneration and tissue remodeling.

Cardiovascular diseases are the leading cause of mortality worldwide, often necessitating long-term, continuous pharmacological intervention. Traditional treatment approaches face challenges such as uneven drug release, low targeting efficiency, and drug-related side effects [55]. Micro-nanomaterials hold promise for overcoming these challenges by enhancing drug delivery methods, thereby improving therapeutic efficacy and reducing adverse effects (Figure 3).

#### 3.3.1. Sustained and Controlled Release of Drugs

Micro-nanomaterials enable the sustained and controlled release of therapeutic agents, prolonging their effects and reducing the frequency of administration. Common sustained-release systems, such as PLGA nanoparticles, can be engineered to regulate the degradation rate of the carrier material, allowing for continuous drug release, maintaining stable drug concentrations in the bloodstream, and improving patient compliance [56,57]. Additionally, magnetic nanoparticles offer the potential for controlled drug release through external magnetic fields, paving the way for personalized therapy [58,59].

#### 3.3.2. Targeted Delivery of Antithrombotic and Anti-Inflammatory Drugs

Targeted delivery of antithrombotic and anti-inflammatory drugs is crucial in the management of cardiovascular diseases. Micro-nanomaterials can enhance therapeutic efficacy and reduce side effects by delivering these drugs specifically to affected areas [60,61,62]. Nanoparticles can also be functionalized with anti-inflammatory molecules to target arterial plaques, thereby reducing inflammation and preventing plaque rupture [63].

#### 3.3.3. Targeted Atherosclerosis Therapy and Myocardial Repair

Atherosclerosis, characterized by lipid deposition in arterial walls, smooth muscle cell proliferation, and fibrosis, is a chronic inflammatory condition that leads to cardiovascular diseases [64]. Micro-nanomaterials can facilitate precise drug delivery through the modification of multifunctional carriers. For instance, nanoparticles, nanoshells, and metal–organic frameworks (MOFs) can be functionalized with specific ligands such as antibodies, peptides, or oligonucleotides to target atherosclerotic plaques, enhancing therapeutic efficacy and reducing systemic side effects [65,66,67,68,69].

In myocardial repair, micro-nanomaterials also play a vital role. Following myocardial infarction, the death of cardiomyocytes and subsequent fibrosis severely impair cardiac function. Micro-nanomaterials can be utilized not only for the delivery of drugs and genes to promote cardiomyocyte regeneration but also for the synthesis of three-dimensional scaffold materials through self-assembly or templating methods [70,71,72]. These scaffolds provide a microenvironment akin to the natural extracellular matrix (ECM), facilitating cell adhesion, proliferation, and differentiation [73,74]. Additionally, nanomaterials enable the controlled release of growth factors or other regenerative signaling molecules, further promoting the repair and remodeling of myocardial tissue.

## 4. Applications of Micro-Nanomaterials in Diagnostics

**Figure 4:**
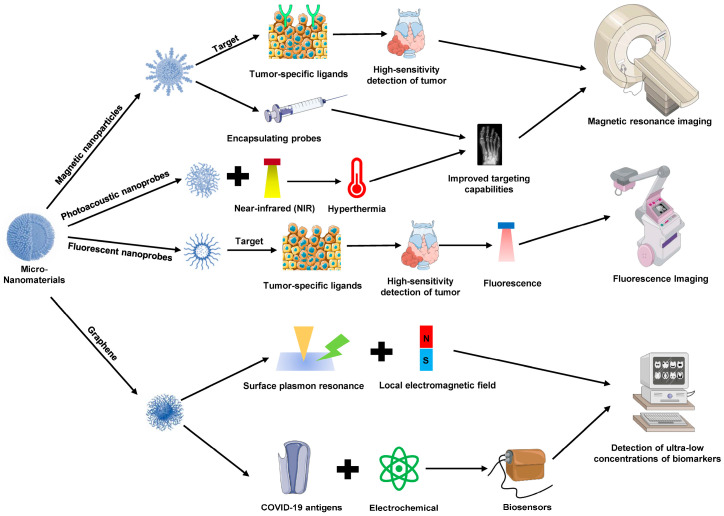
Applications of micro-nanomaterials in diagnostics. In molecular imaging, micro-nanomaterials enhance contrast and resolution in MRI, photoacoustic imaging, and fluorescence imaging. Superparamagnetic iron oxide nanoparticles (SPIONs) improve MRI sensitivity with lower toxicity, while photoacoustic and fluorescent nanoprobes offer high-resolution multimodal imaging, aiding early tumor localization. Additionally, micro-nanomaterials enhance biosensing technologies. In surface plasmon resonance (SPR) and electrochemical biosensors, nanomaterials like gold nanoparticles and graphene improve sensitivity and enable rapid point-of-care diagnostics, including for infectious diseases such as COVID-19.

The application of micro-nanomaterials in diagnostic medicine has become a focal point of contemporary research. Owing to their unique physicochemical properties, these materials exhibit immense potential in enhancing diagnostic accuracy, sensitivity, and early detection. Through functional design, micro-nanomaterials can specifically bind to biomolecules, facilitating early detection and precise diagnosis of diseases using various imaging and sensing technologies (Figure 4).

### 4.1. Applications in Molecular Imaging

Molecular imaging is a cutting-edge field in medical imaging, focused on visualizing and quantifying specific biological processes in vivo to enable early disease detection, monitoring, and treatment evaluation. Although traditional imaging techniques are widely used clinically, their limitations in sensitivity, resolution, and specificity constrain their effectiveness in early disease detection [75]. The integration of micro-nanomaterials with molecular imaging technologies significantly enhances imaging contrast, resolution, and specificity, thereby advancing the development of precision medicine [76,77,78].

#### 4.1.1. Applications in Magnetic Resonance Imaging

Magnetic resonance imaging (MRI) is a non-invasive imaging technology widely used in clinical diagnostics. However, traditional MRI contrast agents, such as gadolinium-based agents, are associated with nephrotoxicity and other adverse effects while enhancing imaging contrast [79]. Magnetic nanoparticles, particularly superparamagnetic iron oxide nanoparticles (SPIONs), have emerged as ideal alternatives to conventional contrast agents due to their favorable magnetic properties and biocompatibility [80]. Through surface functionalization, SPIONs can be modified with tumor-specific ligands (e.g., tumor cell surface receptor-targeting molecules) to achieve high-sensitivity detection of small tumor lesions. In addition to SPIONs, other nanomaterials encapsulating Gd(III)-, Fe(III)-, or Mn(II)-based probes have also been developed to improve MRI contrast. These agents offer enhanced relaxivity and improved targeting capabilities when combined with nanocarriers, reducing toxicity concerns associated with free metal ions. Such nanoprobes enable high-resolution imaging and have been employed in both cancer diagnostics and monitoring of other diseases, providing a versatile platform for safer and more effective MRI contrast enhancement [81]. These functionalized magnetic nanoparticles significantly enhance signal intensity in tumor regions during MRI scans, enabling early and precise tumor localization [82,83].

#### 4.1.2. Applications in Photoacoustic Imaging

Photoacoustic imaging is an emerging technology that utilizes the photoacoustic effect, wherein laser-induced ultrasound signals enable high-resolution imaging of deep tissues. Micro-nanomaterials enhance PAI by amplifying the photoacoustic signal and improving imaging contrast. For instance, photoacoustic nanoprobes possess excellent light absorption properties. In the near-infrared region, these nanoprobes efficiently convert light energy into heat, generating strong photoacoustic signals [84,85]. By surface-modifying these nanoprobes with tumor-targeting molecules, high-contrast imaging of tumors can be achieved. Moreover, combining PAI with nanomaterials facilitates multimodal imaging (e.g., photoacoustic–fluorescence imaging, photoacoustic–MRI imaging), allowing for comprehensive disease assessment from multiple dimensions and further improving diagnostic accuracy [86,87,88].

#### 4.1.3. Applications in Fluorescence Imaging

Fluorescence imaging, known for its high sensitivity and real-time imaging capabilities, is widely utilized in molecular imaging. Functional modification of fluorescent nanoprobes further enhances imaging specificity and multifunctionality. For example, by conjugating targeting peptides or antibodies to the surface of quantum dots, precise identification and imaging of specific tumor markers can be achieved [89]. Upconversion nanoparticles, when combined with photosensitizers, can serve as dual-functional probes for both photodynamic therapy and fluorescence imaging, enabling deep tumor imaging and the generation of reactive oxygen species under light exposure to kill tumor cells, achieving integrated diagnosis and therapy [90,91,92]. In addition to traditional fluorescent probes, self-fluorescent nanomaterials, which do not require external fluorophores, have garnered attention due to their inherent stability, reduced photobleaching, and lower cytotoxicity. These materials enable long-term, real-time imaging in biological systems without the need for additional labeling agents [93].

### 4.2. Biosensing and Point-of-Care Diagnostics

As the spectrum of diseases evolves and diagnostic needs increase—particularly for early screening of major infectious diseases and cancers—rapid, sensitive, and specific diagnostic technologies have become increasingly critical. The application of micro- and nanomaterials in biosensors and point-of-care diagnostics has significantly advanced the capability to meet these needs, providing powerful tools for early disease diagnosis and monitoring.

#### 4.2.1. Biosensors Based on Surface Plasmon Resonance

Surface plasmon resonance (SPR) is an optical sensing technique that detects target molecules in real time by monitoring refractive index changes due to biomolecular interactions on the sensor surface [94]. The incorporation of micro-nanomaterials, particularly gold nanoparticles, silver nanoparticles, and graphene, significantly enhances the sensitivity and detection range of SPR sensors. Gold nanoparticles, with their unique plasmonic properties, can bind to the sensor surface, enhancing the local electromagnetic field, thereby enabling the detection of ultra-low concentrations of biomarkers (e.g., cancer markers, viral antigens) [95,96].

#### 4.2.2. Electrochemical Biosensors

Electrochemical biosensors detect target substances by analyzing the electrical signals generated by the interaction between biomolecules and the electrode surface. These biosensors offer excellent portability and cost-effectiveness, making them ideal for point-of-care testing (POCT). When combined with microfluidic chip technology, electrochemical biosensors incorporating micro- and nanomaterials can achieve rapid multiparameter detection, providing an ideal solution for emergency care, home monitoring, and healthcare in resource-limited settings [97,98]. For example, graphene-based sensors have been successfully applied in the rapid detection of COVID-19 antigens, demonstrating high sensitivity and specificity, significantly reducing detection time and simplifying operational procedures [99].

## 5. Challenges and Strategies for Clinical Translation

The clinical translation of micro-nanomaterials represents a significant leap forward in precision medicine, offering unprecedented opportunities for targeted therapy, advanced diagnostics, and novel therapeutic modalities. However, several critical challenges must be addressed to ensure their safe and effective application in clinical settings [100] (Table 3).

### 5.1. Challenges in Clinical Translation of Nanomaterials for Cancer Theranostics

One of the biggest challenges in the clinical translation of nanomaterials for cancer treatment is ensuring effective tumor targeting while minimizing systemic toxicity. Tumor heterogeneity complicates the targeting of nanoparticles, which are designed to rely on the enhanced permeability and retention (EPR) effect. However, in certain tumors, the EPR effect may be inconsistent or inefficient, leading to suboptimal drug delivery. Moreover, nanoparticles designed for theranostics often face issues with biocompatibility and the body’s immune response [101]. For example, inorganic materials like gold nanoparticles, although useful for imaging and photothermal therapy, may accumulate in organs such as the liver and kidneys, potentially causing toxicity. Similarly, mesoporous silica nanoparticles (MSNs), while effective for drug delivery and imaging, may pose risks of long-term accumulation and inflammation [102].

To address these challenges, various strategies have been developed. One approach involves surface modifications, such as coating nanoparticles with biocompatible polymers like polyethylene glycol (PEG) or using biomimetic coatings, like cloaking with cell membranes, to reduce immune clearance and extend circulation time. Another strategy focuses on enhancing targeting precision by functionalizing nanoparticles with targeted ligands, such as folate or antibodies, that specifically bind to tumor-associated receptors [103]. Additionally, stimuli-responsive systems have been designed, where smart nanoparticles release their therapeutic payload in response to environmental triggers like pH, temperature, or tumor-specific enzymes, thereby improving drug release accuracy and minimizing off-target effects [104].

### 5.2. Challenges in Cardiovascular Applications of Nanomaterials

In cardiovascular diseases, a major challenge is achieving the controlled release of therapeutic agents over extended periods, which is critical for preventing heart attacks or managing chronic conditions like atherosclerosis. Nanoparticles such as PLGA-based systems offer promise for controlled release, but they face challenges in maintaining stable drug concentrations due to rapid degradation or premature drug release. Moreover, achieving targeted delivery to atherosclerotic plaques, while avoiding uptake by other cells like macrophages, remains difficult [105].

To address these challenges, several innovative solutions have been proposed. One involves the development of sustained-release systems using advanced biodegradable materials like PLGA, which offer more controlled degradation and drug release [106]. Magnetically responsive nanoparticles also enable the external regulation of drug release through magnetic fields. In the realm of targeted atherosclerosis therapy, nanoparticles functionalized with molecules that bind to proteins involved in plaque formation can significantly enhance treatment specificity and efficacy [107].

### 5.3. Infectious Disease Applications and the Challenges of Drug-Resistant Pathogens

The increasing prevalence of drug-resistant bacteria presents a major challenge in the use of nanomaterials for infectious diseases. Conventional antibiotics are becoming less effective, and while nanoparticles like silver have antimicrobial properties, their long-term safety in humans is still under question, particularly with regard to their potential toxicity and accumulation.

To address these challenges, several proposed solutions focus on the development of multifunctional nanomaterials. By designing nanomaterials that combine drug delivery with photothermal and photodynamic therapies, antibacterial efficacy can be enhanced while reducing the required dosage of nanomaterials, thereby lowering toxicity risks [108]. Additionally, the use of biocompatible materials, such as polymers or gold nanoparticles, is emphasized to avoid the potential long-term risks associated with toxic metal nanoparticles [109]. Furthermore, targeted design strategies, involving surface functionalization, aim to direct nanomaterials specifically to infection sites, minimizing the impact on healthy tissues [110].

## 6. Clinical Trials and Future Perspectives

### 6.1. Clinical Trials of Microscale and Nanoscale Materials in Theranostics

Nanotechnology-based approaches are at the forefront of precision medicine, and several clinical trials have been launched to test their efficacy and safety in theranostic applications. Below are key examples of ongoing or completed clinical trials focusing on nanomaterials in cancer, cardiovascular diseases, and infectious diseases.

Nanomaterial-based cancer therapy represents one of the most advanced areas in clinical research, offering enhanced drug delivery, improved targeting, and the integration of therapeutic and diagnostic capabilities (theranostics). Several clinical trials have explored these innovations. The Phase III MPACT trial (NCT00844649) investigated the efficacy of nab-paclitaxel (albumin-bound paclitaxel) combined with gemcitabine versus gemcitabine alone in patients with metastatic pancreatic cancer. This trial demonstrated significant improvements in both overall survival (OS) and progression-free survival (PFS), leading to the approval of nab-paclitaxel plus gemcitabine as a standard treatment in 2013 [111,112]. Another trial, NCT01679470, focused on AuroLase therapy, a photothermal treatment for primary and metastatic lung tumors using gold nanoshells (AuroShell^®^). The gold nanoshells accumulated in tumors after being injected into the bloodstream and were heated by near-infrared (NIR) laser light to induce hyperthermia, selectively destroying cancer cells with minimal damage to surrounding tissue. However, this trial was terminated before completion [113].

Nanomaterials are being actively tested in both cardiovascular and infectious diseases for their ability to enhance drug delivery and promote tissue regeneration. In the cardiovascular field, the NANOM-FIM trial (NCT01270139) investigated the safety and efficacy of nanoparticle-based therapies for conditions such as atherosclerosis, heart failure, and stable angina. This trial involved the use of silica–gold iron-bearing nanoparticles combined with stem cells, delivered to atherosclerotic plaques via minimally invasive cardiac surgery in patients with multivessel coronary artery disease. The results demonstrated a significant reduction in atheroma volume and improved cardiovascular outcomes over a 12-month follow-up period, marking a significant advancement in nanoparticle-based treatments for atherosclerosis and related cardiovascular conditions [114,115]. In parallel, the need for novel antimicrobial therapies has driven the exploration of nanomaterials for more effective antibiotic delivery. One notable trial in infectious diseases, NCT04368728, sponsored by Pfizer and BioNTech, evaluated the BNT162 mRNA vaccine candidates against COVID-19. This Phase I/II/III study, which began in April 2020, assessed the safety, tolerability, immunogenicity, and efficacy of the vaccine. The trial played a pivotal role in the development of the BNT162b2 vaccine, later named Comirnaty, which was shown to be approximately 95% effective in preventing symptomatic COVID-19 and became one of the first vaccines authorized for emergency use [116,117,118,119].

### 6.2. Future Perspectives

The future of microscale and nanoscale materials in theranostics is promising but faces several key challenges that must be addressed to achieve widespread clinical use. Below are some of the most critical areas for future research and development.

Despite advancements in the functionalization of nanoparticles for specific targeting, achieving consistent targeting across different patient populations remains a challenge. Tumor heterogeneity, for example, can result in the variable expression of receptors such as folate or HER2, which are commonly used in nanoparticle targeting. Future research should focus on developing nanoparticles that can adapt to the dynamic nature of disease progression, potentially through the use of artificial intelligence (AI)-driven diagnostic tools that personalize treatment in real time. As theranostics continues to evolve, the integration of nanomaterials with personalized medicine platforms will become increasingly important. The ability to diagnose and treat diseases based on a patient’s unique genetic and molecular profile will allow for more effective, individualized treatments. Nanomaterials can play a key role in this by serving as both diagnostic tools and therapeutic delivery vehicles.

Regulatory approval remains a significant hurdle for the clinical translation of nanomedicines. Nanoparticles face unique challenges in terms of manufacturing consistency, scalability, and quality control. Establishing standardized guidelines for the production and testing of nanomaterials is essential. Moreover, clinical trials must be designed to address the specific pharmacokinetic and safety challenges associated with nanomaterials, which differ from conventional small-molecule drugs. In conclusion, microscale and nanoscale materials are poised to revolutionize the field of theranostics, offering unprecedented precision in both diagnostics and treatment. However, several challenges remain, particularly in the areas of targeting accuracy and regulatory approval. As future research continues to address these issues, the integration of nanomaterials into personalized medicine will likely become a key driver of innovation in healthcare.

## 7. Conclusions

In conclusion, microscale and nanoscale materials have revolutionized the landscape of theranostics by offering a highly versatile platform for integrating therapeutic and diagnostic functions. These materials, with their unique physicochemical properties, enable enhanced targeting, controlled drug release, and real-time monitoring of treatment efficacy, particularly in applications such as cancer, cardiovascular diseases, and infectious diseases. Despite the significant advancements demonstrated in preclinical studies, the translation of these promising technologies into clinical practice faces several key challenges. Issues such as biocompatibility, potential toxicity, and complex pharmacokinetics remain critical barriers to overcome. Additionally, achieving regulatory approval is a complex process that requires addressing concerns regarding manufacturing consistency, scalability, and long-term safety.

Clinical trials on nanomaterials for theranostics, such as those involving gold nanoparticles for photothermal therapy and polymer-based nanoparticles for drug delivery, offer promising results, but further research is necessary to refine these technologies for broader clinical adoption. Future efforts should focus on improving the targeting precision, biocompatibility, and clearance of these materials while also addressing regulatory hurdles. The integration of artificial intelligence and personalized medicine platforms will likely enhance the specificity and efficacy of nanomaterial-based treatments, paving the way for more individualized and effective healthcare solutions. As research continues to advance, microscale and nanoscale materials are poised to play a pivotal role in the future of precision medicine, offering new hope for more effective and safer therapeutic interventions.

## Figures and Tables

**Table 1:** Classification and characteristics of micro-nanomaterials for theranostic applications.

Classification	Subclassification	Characteristics	Theranostic Applications
Organic materials	Polymer nanoparticles	Biocompatibility, biodegradability, and ability to encapsulate both hydrophilic and hydrophobic drugs	Therapeutic agents and imaging probes
	Liposomes	Highly biocompatible	Functionalized with targeting ligands that bind to tumor-specific markers
	Dendrimers	Hyper-branched macromolecules	High loading capacity makes them ideal for theranostics.
Inorganic materials	Gold nanoparticles	Unique optical properties	Be conjugated with drugs or imaging agents
	SPIONs	Magnetic properties	Drug delivery and precise tracking through MRI
	MSNs	High surface area and tunable pore sizes	Simultaneous loading of drugs and imaging agents
Composite materials	-	Combine the advantages of both organic and inorganic materials	Facilitated both the therapeutic and diagnostic capabilities within a single platform

**Table 2:** Functionalization of materials and optimization of theranostic systems.

Classification	Mechanism	Applications	Reference
Targeting and Imaging	Be functionalized with ligands that target specific receptors overexpressed on diseased cells	Targeted cancer therapy and real-time fluorescence imaging	[19,20]
	Functionalized with diagnostic probes	Provide visual feedback on drug distribution and efficacy during therapy	[21]
Stimuli-Responsive Systems	Release drugs specifically in acidic environments	Targeting cancer and providing feedback on drug release locations	[22]
	Responded to the target enzyme		[23]
Smart Responsive Systems	Strong absorption in the near-infrared region	Disrupting cancer cells and enabling concurrent real-time imaging	[24]
	Undergoing phase changes at specific temperatures	Providing real-time feedback and enhancing therapeutic outcomes	[25]

**Table 3:** Challenges and strategies for clinical translation.

Classification	Challenges	Strategies
Cancer Theranostics	Ensuring effective tumor targeting	Enhancing targeting precision by functionalizing nanoparticles
	Biocompatibility	Surface modifications, such as coating nanoparticles with biocompatible polymers
	Risks of long-term accumulation and inflammation	
Cardiovascular Theranostics	Controlled release of therapeutic agents over extended periods	Using advanced biodegradable materials and magnetically responsive nanoparticles
Infectious Disease Theranostics	Long-term safety in humans	Designing nanomaterials that combine drug delivery with photothermal and photodynamic therapies

## Abbreviations

siRNA	small interfering RNA
PLGA	poly(L-lactic-co-glycolide)
pH	Pondus Hydrogenii
PTT	photothermal therapy
PDT	photodynamic therapy
MOFs	metal–organic frameworks
ECM	extracellular matrix
EPR	enhanced permeability and retention
MRI	magnetic resonance imaging
SPIONs	superparamagnetic iron oxide nanoparticles
PAI	photoacoustic imaging
SPR	surface plasmon resonance
PEG	polyethylene glycol
